# Sensitive and rapid detection of *tet(X2)* ~ *tet(X5)* by loop-mediated isothermal amplification based on visual OTG dye

**DOI:** 10.1186/s12866-023-02944-4

**Published:** 2023-11-06

**Authors:** Guiling Chen, Lulin Chen, Sisi Lin, Congzhu Yang, Huanlin Liang, Kuang Huang, Zhusheng Guo, Fei Lv

**Affiliations:** 1 Department of Clinical Laboratory, DongGuan SongShan Lake Tungwah Hospital, Dongguan, Guangdong China; 2 Department of Clinical Laboratory, DongGuan Tungwah Hospital, Dongguan, Guangdong China

**Keywords:** Tet(X2/X3/X4/X5), Tigecycline resistance, LAMP, Rapid Detection

## Abstract

The emergence of tigecycline-resistant tet(X2/X3/X4/X5) genes poses a new threat to the efficacy of anti-infective therapy and the safety of our food and environment. To control the transfer of such genes, a sensitive and rapid molecular method is warranted to detect tet(X2/X3/X4/X5) genes in clinical isolates. Herein, we established a loop-mediated isothermal amplification (LAMP) assay to rapidly detect tet(X2/X3/X4/X5) genes, and the results were assessed by chromogenic visualization. The specificity and sensitivity of the primers during the LAMP assay for the simultaneous detection of tet(X2/X3/X4/X5) genes were determined in this study. All 48 clinical strains without tet(X2/X3/X4/X5) genes yielded negative results during the LAMP assay, substantiating the high specificity of the LAMP primers. The detection thresholds of this assay were 1.5 × 10^2^ CFU/ml and 0.2 fg/uL corresponding to a 10 to 100-fold and 100-fold increase in sensitivity compared to polymerase chain reaction (PCR) assays. Out of 52 bacterial strains tested, using PCR as a reference, our research revealed that the LAMP assay demonstrated a sensitivity and specificity of 100%. To sum up, our novel approach has huge prospects for application in the simultaneous detection of tet(X2/X3/X4/X5) genes and can be applied to detect other drug-resistance genes.

**Supplementary Information:**

The online version contains supplementary material available at 10.1186/s12866-023-02944-4.

## Introduction

Tigecycline is widely acknowledged to be a 9-t-butylglycylamido derivative of minocycline. The chemical modification at the C-9 position in the D-loop results in enhanced binding to targets and more effective evasion from common tetracycline resistance mechanisms compared to earlier tetracyclines (tetracycline, doxycycline, and minocycline) [1]. Thus, tigecycline displays broad-spectrum antimicrobial activity against multidrug-resistant (MDR)/extensive drug-resistant (XDR) organisms and is one of the antibiotics of last resort for treating complex infections evoked by MDR gram-negative and gram-positive pathogens [2]. However, bacterial resistance has emerged following the extensive application of tigecycline. In 2019, *tet(X3)* and *tet(X4)*, a novel plasmid-mediated tigecycline resistance mechanism-tetracycline modifying enzyme, were reported in *Enterobacteriaceae* and *Acinetobacter* in China, which showed 85.1 and 94.3% homology to *tet(X)*, respectively [3]. A plasmid-mediated *tet(X5)* was subsequently reported, showing 90.0%, 84.5%, and 90.5% homology to *tet(X2)*, *tet(X3)*, and *tet(X4)* [4]. It has been established that *tet(X3/X4/X5)* confers high-level resistance to tigecycline in bacteria and is not only located in conjugative plasmids (IncFIB IncFII, IncFIA, IncHIA and IncHIB replicon types) [3, 5, 6], but also found adjacent to insertion sequences (ISs) [3, 4, 7], which mediate the horizontal transfer of resistance between different strains and genera. Additionally, *tet(X2)* (1167 bp) on the chromosome (almost identical to *tet(X)* (99.8%)) and *tet(X3/X4)* on plasmids have been detected in clinical samples from Zhejiang Hospital, China, between 1994 and 2019 indicating the potential endemicity of *tet(X2/X3/X4)* in China [8].

Over the years, *tet(X3/X4/X5)* genes on plasmids have been documented in different ecological niches, including livestock, environment, and meat for consumption. Current evidence suggests that tet(X2/X3/X4/X5) is most common in China, with an escalation in annual reports representing a formidable challenge to human health and food security [7–10]. Therefore, a convenient, rapid, and simple method suitable for various applications is urgently required to detect tet(X2/X3/X4/X5) genes in various samples.

In recent years, a multitude of molecular methods have been developed for the detection of *tet(X)* and its mutants, such as RNA-based antibiotic susceptibility testing (RBAST) [11], multiplex polymerase chain reaction (PCR) [12], MALDI-TOF MS [13], the tetracycline inactivation method assay (TIM) [9], a TaqMan-based multiplex real-time PCR assay [14], a multiplex real-time SYBR green-based PCR assay [15], and combined with PCR and Sanger sequencing [16], etc. Although these methods exhibit enhanced detection in terms of rapidity, sensitivity, and specificity, they may not be suitable for large-scale applications given their complexity to operate, dependence on specialized and expensive equipment, lengthy detection time, and high costs. Therefore, another rapid and simple assay, loop-mediated isothermal amplification (LAMP), has been developed to complement the existing PCR methods [17].

LAMP is a nucleic acid detection technique first reported in 2000 that can amplify DNA with Bst DNA polymerase under isothermal conditions and allow auto-cycling strand displacement DNA synthesis. This method can recognize 6–8 different sequences in the target DNA using 4–6 primers, contributing to high specificity and amplification efficiency and its wide application for pathogen detection in infectious diseases [18]. LAMP includes turbidimetry, HNB dye, SYBR green dye, fluorometry, and visual OTG (orange to green) dye, of which HNB dye and SYBR green dye are the most commonly used. Indeed, it is less accurate to judge the results of the turbidimetric method by the naked eye. Accordingly, a turbidimeter is often required to determine the change in turbidity, with a theoretical detection threshold of 10 to 100 copies. It is well-established that the HNB dye and SYBR green dye methods are complicated to operate since they require the manual addition of enzymes, buffers, and dNTPs. Indeed, the cover must be open, and the HNB and SYBR green dyes are added at the end of the reaction, which can easily result in aerosol contamination, giving a theoretical detection limit of 10 to 50 copies. Fluorometry has the highest sensitivity but requires specialized instruments to read fluorescence values, with a theoretical detection limit of one copy. In recent years, an easy-to-operate visual OTG dye kit with premix on enzymes, buffers, and dNTPs has been developed, only requiring the addition of primers and DNA templates. Additionally, the OTG dye is coated on the tube cap, and after the LAMP reaction, the results can be visually interpreted by mixing the product with the tube cap dye, yielding a theoretical detection limit of 10 copies [19, 20].

Herein, a LAMP method based on visual OTG dye was designed and developed for the simultaneous detection of tet(X2/X3/X4/X5) in this study. We optimized the sample processing procedures, which enabled us to complete the whole process from sample processing to result reading within 1 h and accurately interpret the results by the naked eye based on a color change; it was easy to operate, only requiring thermostatic equipment, and suitable for large-scale testing such as community hospitals.

## Materials and methods

### Bacterial strains and preparation of templates

In this study, A total of 48 bacterial strains were obtained from different departments at the DongGuan SongShan Lake Tungwah Hospital (Dongguan, Guangdong, China). These samples were obtained from 2021 to 2022. The 48 bacterial strains without the tet(X2/X3/X4/X5) gene were determined by PCR, including pan-resistant *Klebsiella pneumoniae* KPN142 (Efflux pump overexpression-mediated resistance to tigecycline) [21], carbapenem-resistant, tigecycline-resistant hypervirulent *Klebsiella pneumoniae* KPN857-2 (Efflux pump and *tet(A)* overexpression-mediated resistance to tigecycline), multi-drug resistant hypervirulent *Klebsiella pneumoniae* HvKP247 [22], and *Acinetobacter baumannii* Aba912 [23] were used as negative controls. Recombinant bacteria DH5α-pUC57-tet(X2), DH5α-pUC57-tet(X3), DH5α-pUC57-tet(X4), and DH5α-pUC57-tet(X5) were adopted as positive controls (Supplementary Table 1).

### Primer design

**Fig. 1:**
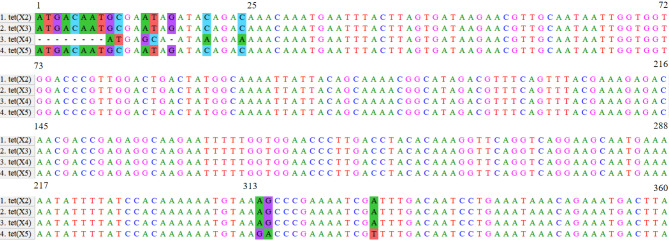
The common conserved sequences of *tet(X2)*, *tet(X3)*, *tet(X4)*, and *tet(X5)* genes at the base loci 25–313

The sequences of *tet(X2)*, *tet(X3)*, *tet(X4)*, and *tet(X5)* genes were retrieved from the GenBank database (accession number: AJ311171.1, MK134375.1, MK134376.1, and CP040912.1); the sequences of *tet(X2)*, *tet(X3)*, *tet(X4)*, and *tet(X5)* genes were aligned using the MEGA-X software. A common conserved sequence was found at the base loci 25–313 with *tet(X2)* as a reference (Fig. 1). Therefore, the Primer Explorer (version 5) software (http://primerexplorer.jp/lampv5e/index.html) was employed to design specific primers, the outer forward/backward primer (F3/B3), forward/backward inner primer (FIP/BIP), additional loop primers (LF/LB) (Table 1). The primers of *tet(X2)*, *tet(X3)*, *tet(X4)*, and *tet(X5)* used for PCR were based on previous reports (Table 1). The *tet(X2)*, *tet(X3)*, *tet(X4)*, and *tet(X5)* gene sequences and all primers were synthesized by Sangon Biotech Co., Ltd. (Shanghai, China).

**Table 1:** The primers of LAMP assay and PCR assay for *tet(X2)*, *tet(X3)*, *tet(X4)*, and *tet(X5)* genes

Gene	Primer	Sequence (5′−3′)	*T*_*m*_ (℃)	size (bp)	Reference
*tet(X2/X3/X4/X5)*	F3	GTTGGACTGACTATGGCA	51	-	This study
	B3	ACCCATTGGTAAGGCTAAG	51		This study
	FIP	CCTCTCGGTCGTTGTCTCTTTAAATTATTACAGCAAAACGGCA	65		This study
	BIP	CAAGAATTTTTGGTGGAACCCTTGTAAGTTTGTAACAATCCCGCTT	65		This study
	LB	CAAAGGTTCAGGTCAGGAAGCAATG	58		This study
*tet(X2)*	Forward	CGGGATGTCCAAGGTAAGAAAA	54	434	[12]
	Reverse	TGACAACGTCGTATGAATCAA	51		
*tet(X3)*	Forward	GGTATCAACATTTCAATGCTTG	49	265	[12]
	Reverse	CGATTCGTCCTGCGTATCTTTTG	56		
*tet(X4)*	Forward	CTGATTCGTGTGACATCATCTTTTG	53	204	[12]
	Reverse	GTTAAATTTCCCATTGGTCAGATTA	50		
*tet(X5)*	Forward	TGCCGTTGACCTACACAAAGG	57	161	[15]
	Reverse	TGTCAAAACGATTTTCGGGTC	53		

### Preparation of bacterial suspension and DNA extraction

DH5α-pUC57-tet(X2), DH5α-pUC57-tet(X3), DH5α-pUC57-tet(X4), DH5α-pUC57-tet(X5), and 48 bacteria without the tet(X2/X3/X4/X5) gene bacterial suspension at 0.5 McF (approximately 1.5 × 10^8^ CFU/mL) were prepared. Total DNA was extracted using DNA-EZ Reagents V All-DNA-Out (Sangon Biotech Co., Ltd) according to the manufacturer’s instructions, which can be applied to insects, fungi, plants, animals, forensic samples (including whole blood, blood stain, seminal stain, saliva, hair, tissue samples, and buccal cells), paraffin-embedded tissue sections, etc. The prepared bacterial suspension was centrifuged at 11,340 g for 5 min. After removal of the supernatant, the precipitate was re-suspended with the addition of 40 µL sterilized ddH_2_O. Then, 5 µl of resuspension was added to 45 µl of DNA-EZ Reagents and incubated at 80℃ for 5 min for total DNA extraction. Ultimately, 2 µL extracted bacterial suspension was used as a template for LAMP assay, and 1 µL bacterial lysate was adopted as a template for PCR.

Plasmid DNA was obtained from DH5α-pUC57-tet(X2), DH5α-pUC57-tet(X3), DH5α-pUC57-tet(X4), DH5α-pUC57-tet(X5) using the TaKaRa MiniBEST Plasmid Purification Kit Ver.4.0 (Takara) according to the manufacturer’s instructions. Ultimately, 2 µL plasmid DNA was used as a template for LAMP assay, and 1 µL plasmid DNA was adopted as a template for PCR.

### LAMP assay

The Lyophilized LAMP Kit (Visual OTG Dye, Beijing Baiao Laibo Technology Co., Ltd, Beijing, China) was adopted for LAMP assay. The LAMP assay was carried out in 20 µL reaction mixtures containing the following components: 15 µL LAMP OTG Reagent, 3 µL LAMP Primer Mix, and 2 µL DNA template. LAMP Primer Mix concentration: 2 µM F3/2 µM B3, 16 µM FIP/16 µM BIP, 8 µM LF/8 µM LB. The LAMP assay was implemented with a reaction temperature of 65℃ and a reaction time of 45 minus per the manufacturer’s instructions. The results could be interpreted by the naked eye, with a positive result indicated by a color change from orange to green and a negative result indicated by the color remaining orange. The experiment was repeated three times to ensure accuracy and consistency.

### PCR assay

The PCR assay was conducted to compare the sensitivity of the LAMP assay for *tet(X2)*, *tet(X3)*, *tet(X4)*, and *tet(X5)* gene detection using the reported primers. The PCR assay was performed in 50 µL reaction mixtures containing the following components: 25 µL 2X SanTaq PCR Mix (Sangon Biotech Co., Ltd), 1 µL DNA template, 1 µL forward primer (10 µmol/L), 1 µL reverse primer (10 µmol/L), sterilized ddH_2_O supplemented to 50 µL. The reaction conditions consisted of pre-denaturation at 94℃ (5 min), denaturation at 94℃ (30 s), annealing at *T*_*m*_ (30 s), extension at 72℃, 30 s), and final extension at 72℃ (7 min), 30 cycles in total. PCR products were analyzed by 1.5% agarose gel electrophoresis.

### Spiked urine specimens, blood specimens, and cerebrospinal fluid specimens

An amount of 1.5 × 10^8^ bacteria of DH5α-pUC57-tet(X2), DH5α-pUC57-tet(X3), DH5α-pUC57-tet(X4), and DH5α-pUC57-tet(X5) were spiked in 1000 µL of clinical specimens (including urine specimens, blood specimens, and cerebrospinal fluid specimens), respectively. Consequently, the manipulation of the sample was validated by relevant literature [19]. The sediment of these samples was mixed and re-suspended with 50 µL of DNA-EZ reagent, then 2 µL extracted bacterial suspension was subjected to the LAMP assay template [24].

## Results

### Specificity and sensitivity of the LAMP assay for tet(X2/X3/X4/X5)

The 48 bacterial strains and blank controls were used as negative controls to test the specificity of the LAMP assay for the *tet(X2)*, *tet(X3)*, *tet(X4)*, and *tet(X5)* genes, respectively. To evaluate the sensitivity of LAMP Assay for the detection of *tet(X2)*, *tet(X3)*, *tet(X4)*, and *tet(X5)* genes, the present study used DH5α-pUC57-tet(X2), DH5α-pUC57-tet(X3), DH5α-pUC57-tet(X4), and DH5α-pUC57-tet(X5) as positive controls. Extracted bacterial suspension and plasmid DNA were assayed in a 10-fold dilution series ranging from 1.5 × 10^6^ CFU/ml to 1.5 × 10^1^ CFU/ml and from 2 pg/µL to 0.02 fg/µL, respectively.

**Fig. 2:**
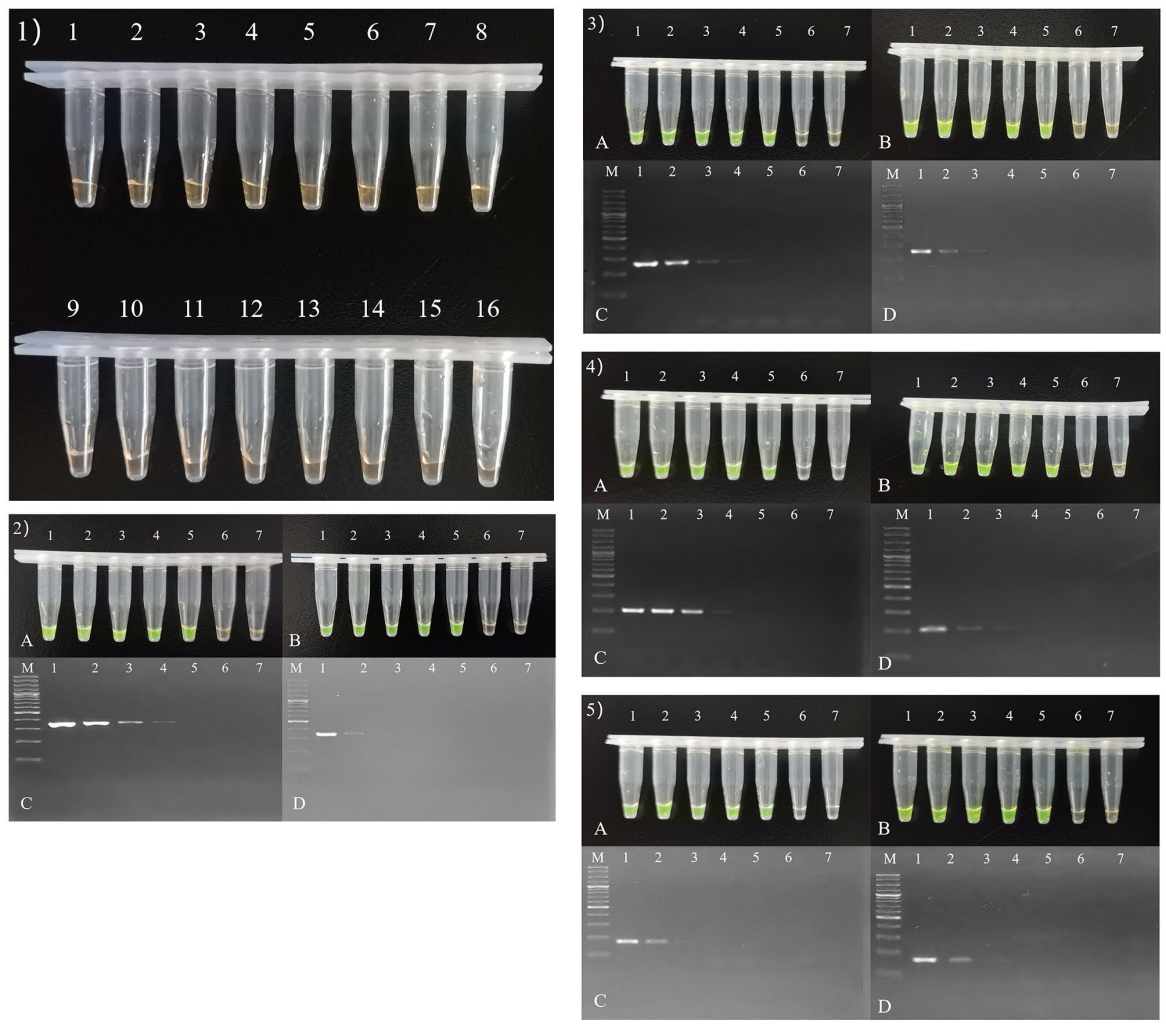
1) LAMP assay specificity for tet(X2/X3/X4/X5) among 16 of 48 bacteria without the tet(X2/X3/X4/X5) gene. 2–5) sensitivities of LAMP assay (**A** and **B**) and PCR (**C** and **D**) for tet(X2/X3/X4/X5). Lane M, Ladder H1-K (100 ~ 3000 bp) DNA marker. In **A** and **C**, lane 1–7, serial 10-fold dilutions of bacterial lysates from 10^6^ CFU/mL to 10^1^ CFU/mL. Lane 8, negative (water). In **B** and **D**, lane 1–7, serial 10-fold dilutions of plasmid DNA from 2 pg/µL to 0.02 fg/µL. Lane 8, negative (water)

As shown in Fig. 2, the LAMP assays yielded positive results only when DH5α-pUC57-tet(X2), DH5α-pUC57-tet(X3), DH5α-pUC57-tet(X4), and DH5α-pUC57-tet(X5) were used as templates. Importantly, 48 bacteria without the tet(X2/X3/X4/X5) gene (including the blank control) were negative, indicating that the LAMP assay was highly specific for tet(X2/X3/X4/X5).

The thresholds of LAMP assay for DH5α-pUC57-tet(X2), DH5α-pUC57-tet(X3), DH5α-pUC57-tet(X4), and DH5α-pUC57-tet(X5) were 1.5 × 10^2^ CFU/ml, 10 to 100-fold more sensitive than PCR. Besides, the thresholds of LAMP assay for plasmid DNA of pUC57-tet(X2), pUC57-tet(X3), pUC57-tet(X4), and pUC57-tet(X5) were 0.2 fg/µL, 100-fold more sensitive than PCR.

The thresholds of PCR assay for DH5α-pUC57-tet(X2), DH5α-pUC57-tet(X3), DH5α-pUC57-tet(X4), and DH5α-pUC57-tet(X5) were 1.5 × 10^3^ CFU/ml, 1.5 × 10^3^ CFU/ml, 1.5 × 10^3^ CFU/ml, and 1.5 × 10^4^ CFU/ml, respectively. Besides, the thresholds of PCR assay for plasmid DNA of pUC57-tet(X2), pUC57-tet(X3), pUC57-tet(X4), and pUC57-tet(X5) were 20 fg/µL.

The LAMP method was compared to PCR, the gold standard, for 52 samples. The results are presented in Supplementary Table 2. The LAMP assay was employed to identify the presence of tet(X2/X3/X4/X5) genes, which yielded a total of four positive strains (DH5α-pUC57-tet(X2), DH5α-pUC57-tet(X3), DH5α-pUC57-tet(X4), and DH5α-pUC57-tet(X5)) and 48 negative strains. In general, the sensitivity, specificity, positive predictive value (PPV), and negative predictive value (NPV) of LAMP assay to detect tet(X2/X3/X4/X5) genes were 100% (CI: 39.6–100%), 100% (CI: 90.7–100%), 100% (CI: 56.1–100%), and 100% (CI: 90.8–100%), respectively (Supplementary Table 2).

### Evaluation of the lamp assay in spiked urine specimens, blood specimens, and cerebrospinal fluid specimens

LAMP assays were applied to urine specimens, blood specimens, and cerebrospinal fluid specimens spiked with different amounts of tet(X2/X3/X4/X5) bacteria. PCR was also carried out as a control. The results showed that the detection limit of the LAMP assay for urine specimens, blood specimens, and cerebrospinal fluid specimens were 10^2^ CFU/mL, showing 10 to 100-fold and 100-fold higher sensitivity than PCR. Thus, LAMP assays can be efficiently applied to detection of tet(X2/X3/X4/X5) genes in urine specimens, blood specimens, and cerebrospinal fluid specimens.

## Discussion

Tigecycline is regarded as the last resort drug against bacterial infections and is mainly used to treat skin tissue infections, tumors, bacterial pneumonia, and complicated intra-abdominal infections [25]. In addition, tet(X2/X3/X4/X5) is most prevalent in China, with annual increases in its detection rate, posing a great challenge to human health and food security [8, 26].

Lei Xu et al. reported that Plumbagin, an inhibitor of *tet(X3)*/*tet(X4)*, has shown much promise as a lead drug and an adjunct with tetracyclines to treat infections caused by bacteria, especially XDR bacteria harboring *tet(X3)*/*tet(X4)* [27]. Accordingly, the combination of Plumbagin and tigecycline may be used for treatment if the relevant genes are detected in the future. Therefore, we believe there is no need to type tet(X2/X3/X4/X5) because of highly homologous and the same resistance mechanism.

It is well-established that the detection of tet(X2/X3/X4/X5) has significant value. Traditional PCR, multiplex PCR, quantitative fluorescence PCR, and TIM assays have high requirements for specialized instruments and equipment, costs, and laboratories, which are complicated to operate and unsuitable for large-scale detection in primary hospitals such as community hospitals. The present study developed a simple and specificity LAMP assay based on visual OTG dye. Universal primers were designed to simultaneously detect *tet(X2)*, *tet(X3)*, *tet(X4)*, and *tet(X5)* using the LAMP assay; the detection limit was 1.5 × 10^2^ CFU/ml and 0.2 fg/µL, showing 10 to 100-fold and 100-fold higher sensitivity than PCR, respectively.

And when the PCR detection limit is reached, the band is very faint, which can easily lead to false negative. Furthermore, our study optimized the total DNA extraction procedure, offering a theoretical basis for applying the LAMP assay to detect other clinical samples, such as blood. However, a limitation of our study is that tests were not conducted on clinical strains since we did not have a clinical strain harboring tet(X2/X3/X4/X5).

The LAMP assay had demonstrated a sensitivity, specificity, PPV and NPV of 100%, indicating its potential as an effective means of detecting the tet(X2/X3/X4/X5) genes. Although the number of strains tested was limited, we remained confident in the method’s capabilities. Currently, there was a limited amount of literature available on the use of fluorescence PCR for detecting tet(X2/X3/X4/X5). Therefore, fluorescence PCR was not being utilized as a reference in this study. While Li et al. did use fluorescent PCR to detect *tet(X3)* and *tet(X4)* [28], the expression of the test results (copy/µL) was inconsistent, making it difficult to effectively compare. Although traditional PCR was used as a reference in this study, fluorescence PCR offered theoretical detection advantages. Laboratories equipped with professional instruments and equipment were recommended to use fluorescent PCR for detecting the target gene. However, the objective of this study was to target community hospitals lacking professional instruments. Therefore, the LAMP assay established in this study for detecting tet(X2/X3/X4/X5) remained significant.

A recent publication had introduced a new nucleic acids isothermal amplification technology named ladder-shape melting temperature isothermal amplification (LMTIA) that employed one or two pairs of primers and heat-stable DNA polymerase to amplify target genes. While currently focused on food detection, this method had shown comparable or superior sensitivity and specificity to both PCR and LAMP assays, suggesting its potential for future use in detecting microorganisms and drug resistance genes [29–33].

## Conclusion

This study established a highly sensitive and specific LAMP assay for tet(X2/X3/X4/X5) gene detection. The LAMP assay is specific and simple for detecting other drug-resistance genes, showing advantages over traditional PCR, turbidimetry, HNB dye, and SYBR green dye. This approach has huge prospects for application in the routine screening of clinical drug-resistant bacteria. To our knowledge, this study is the first to use LAMP technology based on visual OTG dye to simultaneously detect tet(X2/X3/X4/X5) genes.

### Electronic supplementary material

Below is the link to the electronic supplementary material.

Supplementary Material 1

Supplementary Material 2

## Data Availability

The datasets used and/or analysed during the current study are available from the corresponding author on reasonable request.
