# Supplementary material for: Sensitive and rapid detection of tet(X2) ~ tet(X5) by loop-mediated isothermal amplification based on visual OTG dye
Source: BMC Microbiol. 2023 Nov 6;23:329. doi: 10.1186/s12866-023-02944-4 (PMC10626792; doi:10.1186/s12866-023-02944-4)
Supplement: Supplementary file 1 — Supplementary Material 1 [file 12866_2023_2944_MOESM1_ESM.docx]

Supplementary Table 1 Bacterial strains used in this study

| No. | Species | Characteristic | Source |
| --- | --- | --- | --- |
| 1 | *Klebsiella pneumonia* KPN142 | pan-resistant, tigecycline-resistant | [21] |
| 2 | *Klebsiella pneumonia* HvKP234 | hypervirulent  *Klebsiella pneumoniae* | [22] |
| 3 | *Klebsiella pneumonia* HvKP236 | hypervirulent  *Klebsiella pneumoniae* | [22] |
| 4 | *Klebsiella pneumonia* HvKP246 | hypervirulent  *Klebsiella pneumoniae* | [22] |
| 5 | *Klebsiella pneumonia* HvKP247 | MDR, hypervirulent  *Klebsiella pneumoniae* | [22] |
| 6 | *Acinetobacter baumannii* Aba912 | MDR | [23] |
| 7 | *Escherichia coli* ATCC 25922 | - | Standard strain |
| 8 | *Klebsiella pneumonia*  ATCC 700603 | - | Standard strain |
| 9 | *Pseudomonas aeruginosa*  ATCC 27853 | - | Standard strain |
| 10 | *Klebsiella pneumonia* KPN857-2 | XDR, tigecycline-resistant | Clinical isolate |
| 11 | *Enterobacter cloacae* 916 | MDR | Clinical isolate |
| 12 | *Enterobacter aerogenes* 917 | MDR | Clinical isolate |
| 13 | *Salmonella* 918 | MDR | Clinical isolate |
| 14 | *Serratia marcescens* 919 | MDR | Clinical isolate |
| 15 | *Proteus mirabilis* 920 | MDR | Clinical isolate |
| 16 | *Stenotrophomonas maltophilia* 921 | MDR | Clinical isolate |
| 17 | *Klebsiella pneumonia* 922 | MDR | Clinical isolate |
| 18 | *Klebsiella pneumonia* 923 | MDR | Clinical isolate |
| 19 | *Klebsiella pneumonia* 924 | MDR | Clinical isolate |
| 20 | *Klebsiella pneumonia* 925 | MDR | Clinical isolate |
| 21 | *Klebsiella pneumonia* 926 | MDR | Clinical isolate |
| 22 | *Klebsiella pneumonia* 927 | MDR | Clinical isolate |
| 23 | *Klebsiella pneumonia* 928 | MDR | Clinical isolate |
| 24 | *Klebsiella pneumonia* 929 | MDR | Clinical isolate |
| 25 | *Klebsiella pneumonia* 930 | MDR | Clinical isolate |
| 26 | *Klebsiella pneumonia* 931 | MDR | Clinical isolate |
| 27 | *Klebsiella pneumonia* 932 | MDR | Clinical isolate |
| 28 | *Klebsiella pneumonia* 933 | MDR | Clinical isolate |
| 29 | *Klebsiella pneumonia* 934 | MDR | Clinical isolate |
| 30 | *Klebsiella pneumonia* 935 | MDR | Clinical isolate |
| 31 | *Klebsiella pneumonia* 936 | MDR | Clinical isolate |
| 32 | *Klebsiella pneumonia* 937 | MDR | Clinical isolate |
| 33 | *Escherichia coli* 938 | MDR | Clinical isolate |
| 34 | *Escherichia coli* 939 | MDR | Clinical isolate |
| 35 | *Escherichia coli* 940 | MDR | Clinical isolate |
| 36 | *Escherichia coli* 941 | MDR | Clinical isolate |
| 37 | *Escherichia coli* 942 | MDR | Clinical isolate |
| 38 | *Escherichia coli* 943 | MDR | Clinical isolate |
| 39 | *Escherichia coli* 944 | MDR | Clinical isolate |
| 40 | *Escherichia coli* 945 | MDR | Clinical isolate |
| 41 | *Escherichia coli* 946 | MDR | Clinical isolate |
| 42 | *Escherichia coli* 947 | MDR | Clinical isolate |
| 43 | *Escherichia coli* 948 | MDR | Clinical isolate |
| 44 | *Escherichia coli* 949 | MDR | Clinical isolate |
| 45 | *Escherichia coli* 950 | MDR | Clinical isolate |
| 46 | *Enterobacter cloacae* 951 | MDR | Clinical isolate |
| 47 | *Enterobacter cloacae* 952 | MDR | Clinical isolate |
| 48 | *Enterobacter cloacae* 953 | MDR | Clinical isolate |
| 49 | *Enterobacter cloacae* 954 | MDR | Clinical isolate |
| 50 | *Enterobacter aerogenes* 955 | MDR | Clinical isolate |
| 51 | *Enterobacter aerogenes* 956 | MDR | Clinical isolate |
| 52 | *Enterobacter aerogenes* 957 | MDR | Clinical isolate |
