# Supplementary material for: Sensitive and rapid detection of tet(X2) ~ tet(X5) by loop-mediated isothermal amplification based on visual OTG dye
Source: BMC Microbiol. 2023 Nov 6;23:329. doi: 10.1186/s12866-023-02944-4 (PMC10626792; doi:10.1186/s12866-023-02944-4)
Supplement: Supplementary file 2 — Supplementary Material 2 [file 12866_2023_2944_MOESM2_ESM.docx]

Supplementary Table 2 Comparison of PCR and LAMP assay results on 52 bacterial strains

|  | | PCR | | Sensitivity  % [95% CI] | Specificity  % [95% CI] | PPV  % [95% CI] | NPV  % [95% CI] | K |
| --- | --- | --- | --- | --- | --- | --- | --- | --- |
|  |  | Positive | Negative |  |  |  |  |  |
| LAMP | Positive | 4 (TP) | 0 (FP) | 100 | 100 | 100 | 100 | 1 |
|  | Negative | 0 (FN) | 48 (TN) | 39.6-100 | 90.7-100 | 56.1-100 | 90.8-100 |  |

CI, Confidence Interval; TP, true positive, FP, false positive; TN, true negative; FN, false negative; PPV, positive predictive value; NPV, negative predictive value; K,

Cohen’s Kappa.
